# Luteal phase support in fresh and frozen embryo transfers

**DOI:** 10.3389/frph.2022.919948

**Published:** 2022-07-19

**Authors:** Shirley Greenbaum, Ahlad Athavale, Anat Hershko Klement, Yaakov Bentov

**Affiliations:** ^1^Faculty of Medicine, Hebrew University of Jerusalem, Jerusalem, Israel; ^2^Division of Obstetrics and Gynecology, Hadassah-Hebrew University Medical Center, Jerusalem, Israel; ^3^Department of Obstetrics and Gynecology, Hadassah Mount Scopus-Hebrew University Medical Center, Jerusalem, Israel

**Keywords:** routes of administration, hCG, GnRH agonist, estradiol, progesterone, corpus luteum

## Abstract

**Context:**

Luteal phase support (LPS) has become an essential component of IVF protocols following both fresh and frozen embryo transfers, yet there is still controversy with regards to the optimal protocol of LPS to enhance treatment outcome.

**Search strategy:**

A search *via* PubMed for all the selected topics was limited to publications from the past 10 years and to English language. We subsequently searched the reference lists of retrieved articles. Where available, RCTs were chosen over non-randomized studies. Here we provide an updated review of the current literature on various issues relating to LPS, in both fresh and frozen embryo transfers. The timing of LPS initiation as well as the route of administration and dosing are discussed for both fresh and frozen transfers. A separate discussion for frozen thawed embryo transfer in natural cycles and non-ovulatory cycles is presented.

**Conclusions:**

We present data that supports the use of Progesterone LPS in fresh and frozen embryo transfers. No benefits were found to the addition of hCG or estradiol to progesterone LPS in fresh transfers, however GnRH agonist may have a role. IM Progesterone was not advantageous over vaginal progesterone in fresh transfers but was superior in frozen transfers. The timing of LPS introduction, the interval to embryo transfer, as well as the serum concentration of progesterone, have significant effects on the success of the treatment.

## Introduction

Following ovulation, remnants of the ovulated ovarian follicle form the corpus luteum (CL). This temporal endocrine structure secrets a myriad of hormones such as progesterone, estrogen, relaxin and vasoactive and angiogenic substances (1). The CL is considered an essential component in supporting a healthy pregnancy in its first weeks (2). The most significant product of the CL, progesterone elicits a chain of events resulting in the decidualization of the endometrium and establishment of endometrial receptivity, allowing embryo implantation within a narrow time frame termed the window of implantation (WOI). The administration of the hormonal products of a CL, concurrent with the secretions of an active CL or in its absence, is termed luteal phase support (LPS). LPS can be administrated to patients following controlled ovarian hyperstimulation followed by oocyte retrieval or artificial insemination, or in preparation for a frozen thawed embryo transfer. While pregnancy can develop without exogenous administration of luteal support in natural cycles, it is now well established that luteal support, mainly progesterone, may promote implantation and increase pregnancy success rate in fertility patients (3).

A luteal phase that is shorter than normal or is associated with insufficient progesterone levels is termed luteal phase defect. Luteal phase defect is inherent in non-ovulatory cycles when thawed embryo are transferred in the absence of a corpus luteum, making exogenous luteal support essential. Luteal phase defect is also more likely with controlled ovarian stimulation that may result in supernumerary CL that in combination often secrete high amounts of progesterone, that in turn may elicit strong negative feedback on pituitary LH secretion. This CL derived suppression may lead to a premature drop in LH serum concentration and progesterone withdrawal induced menstrual bleeding (4).

Over the years, to optimize IVF treatment results and patient safety and to reduce discomfort, multiple LPS protocols were developed that are clinically available. Although several systematic reviews and meta-analyses have been published on this topic, multiple important, recently published studies call for an updated summary. This review encompasses an updated summary of the current body of evidence on luteal support in the main treatment protocols. Data were acquired *via* PUBMED search according to the topics addressed, and was limited to the past 10 years and English language. We also manually searched references in previously published reviews and meta-analyses. We will discuss the timing of luteal support, routes of administration, and different combination therapies in several clinical scenarios. We will address separately the administration of LPS in the presence of a functioning CL (i.e., hormonal support treatment), and in non-ovulatory cycles, in the absence of a CL (i.e., hormone replacement therapy).

### Luteal support in fresh embryo transfers

In fresh embryo transfers both estrogen induced endometrial proliferation and progesterone induced endometrial decidualization are byproducts of gonadotropin ovarian stimulation that is administered primarily for the purpose of follicle maturation and the harvesting of oocytes to create embryos. It was therefore not until quite recently that the need for LPS in fresh embryo transfers was appreciated (5, 6). Luteal support in the presence of a CL can be achieved by preventing the premature drop in the LH stimulation of the CL to secrete progesterone, by administering a GnRH agonist or hCG. Alternatively, supplemental progesterone may be given with or without CL stimulation.

### Progesterone and adjuvant LPS

A meta-analysis that included 8 randomized controlled trials and 875 women, compared the administration of luteal progesterone vs. placebo or no treatment (3). The analysis comparing the effects of LPS on ongoing pregnancy rate demonstrated a significant yet mild advantage to progesterone LPS (OR 1.77, 95% 1.09–2.86). However, when only studies that continued progesterone LPS beyond the first pregnancy test and until 12 weeks of pregnancy were included, the positive effects of the LPS became more prominent (OR 2.17 95% 1.37–3.43) (3). Studies that examined the effect of addition of hCG to progesterone failed to show any advantage to using progesterone alone however, the rate of ovarian hyperstimulation syndrome (OHSS) was significantly higher (3). The addition of estrogen to progesterone LPS was analyzed in 9 randomized controlled trials (RCTs) that included several routes of estrogen administration. This meta-analysis failed to demonstrate a positive effect of estrogen co-administration on ongoing pregnancy rate, regardless of the route of estrogen administration (3). However, the addition of GnRH agonist, either in a single dose or repeated administration, was shown to improve ongoing pregnancy rate compared to progesterone alone (OR 0.62, 95% CI 0.48–0.81) without an increased risk for OHSS (3).

#### Progesterone routes of administration in fresh embryo transfers

A Cochrane meta-analysis that compared several routes of progesterone administration in fresh embryo transfer settings demonstrated mixed results (3). The study showed no statistically significant differences between intramuscular (IM) treatment vs. oral (OR 0.71, 95% CI 0.14 to 3.66), or between IM vs. vaginal/rectal (OR 1.37, 95% CI 0.94–1.99). Likewise, two recently published retrospective studies compared combined IM and vaginal progesterone vs. either IM or vaginal progesterone only LPS. Both studies showed similar outcomes for all study arms (7, 8). Similarly, no significant differences were found between low-dose vs. high dose vaginal agents, short vs. long protocol, micronized vs. synthetic formulation or other administration forms (vaginal ring, gel) (3). However, despite these findings, studies in which serum progesterone was measured found serum progesterone concentration that is either too low or too high to be associated with a lower pregnancy rate, suggesting that the LPS dose of progesterone may be important (9, 10). Thomsen et al. prospectively measured serum progesterone in over 600 fresh embryo transfers during the early and mid-luteal stage. The authors found that serum progesterone of 60–100 nmol/L and 150–250 nmol/L during the early and mid-luteal phase, respectively, were associated with the highest pregnancy and live birth rates. Serum progesterone that was either lower or higher than that range correlated with a significantly poorer outcome (10).

#### Timing of progesterone administration in fresh embryo transfers

**Figure 1:**
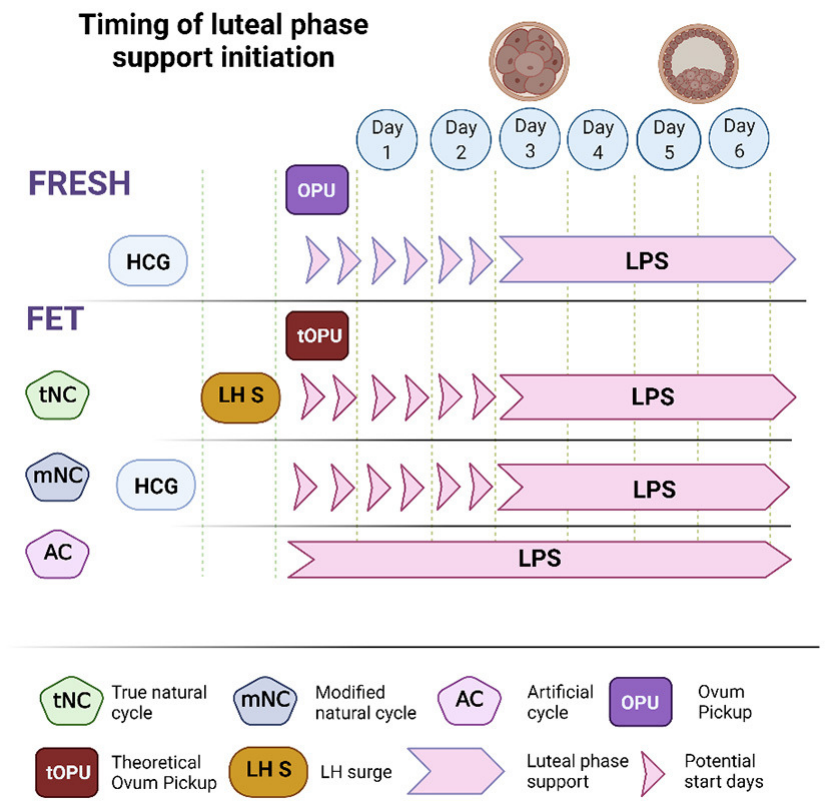
The optimal range of LPS initiation during fresh and frozen embryo transfer. In fresh cycles the optimal range of LPS initiation was defined as day of OPU and up to 3 days later. In frozen transfers, the start of LPS depends on the presence of a corpus luteum (CL). In artificial cycles that take place in the absence of a CL, the transfer of a day 3 embryo and a blastocyst should be performed on the 4^th^ and 6^th^ days of LPS, respectively. In natural and modified natural FETs we refer to a theoretical OPU to standardize the optimal range of LPS initiation. Embryo transfer should be performed 1 day sooner following an LH surge in a tNC, compared to hCG trigger in a mNC.

In fresh cycles, ovulation is accompanied by endogenous progesterone secretion that often originates from multiple CL. In this circumstance, an inaccurate timing of LPS introduction may have significant implications: premature exposure to progesterone may perturb the window of implantation (WOI) while delayed LPS administration might not prevent a premature drop in progesterone and consequentially endometrial shedding. To date, several studies have addressed this question by comparing different timetables for the administration of LPS. Among 5 RCTs that compared different starting times of progesterone administration, only 2 reported statistically significant results (11). Sohn et al. (12) compared administration 12 h prior to ovum pick up (OPU) vs. 24 h after OPU and found a significant advantage to delayed administration (12.9 vs. 24.6%). Williams et al. (13) found significant advantages to starting progesterone LPS on day 3 post OPU rather than delaying it to day 6 (61.0 vs. 44.8%). Taken together, these studies found that initiation of LPS within the time frame of the evening of OPU up until 3 days post OPU, was associated with optimal chances for pregnancy (11, 14) (Figure 1).

#### Personalization of progesterone support in fresh embryo transfers

Determining the optimal dose of progesterone for LPS in the context of fresh ET has generated great interest. A meta-analysis that included 5 studies comparing ongoing pregnancy rate (OPR) or live birth rate (LBR) in patients receiving a low or a high dose of micronized vaginal progesterone (MVP), found no difference in outcome (3). On the other hand, Thomsen et al. that conducted a large non-interventional prospective study analyzing the relationship between early and mid-luteal progesterone serum concentration and treatment outcome, found an optimal range of serum progesterone that was associated with the highest live birth rate. They found that early luteal serum progesterone concentrations of 60–100 nmol/l and mid luteal serum progesterone concentrations of 150–250 nmol/l gave optimal results, whereas serum progesterone concentrations below or above this range were associated with a poorer live birth rate (10).

### LPS following GnRH agonist trigger

The use of a GnRH agonist trigger is often reserved for patients with increased OHSS risk, or in elective freeze-all cycles for which LPS is not necessary. Recently, several publications examined cycles that were triggered with a GnRH agonist to reduce the risk for OHSS, but that subsequently proceeded with a fresh ET. The challenges in designing LPS post GnRH agonist are the higher chances for rapid luteolysis on one hand, and the need to reduce the risk for OHSS on the other (15). Elgindy et al. (16) conducted a RCT comparing IM vs. vaginal progesterone administration in patients with increased baseline OHSS risk that were triggered with either GnRH agonist or 5,000 IU of HCG. Patients triggered with a GnRH agonist received 1,500 IU of hCG on the day of OPU as well as oral estradiol and IM progesterone, while those triggered with hCG received oral estradiol and micronized vaginal progesterone (MVP). The authors reported on a similar ongoing pregnancy rate (OPR). Of note, the rate of considerable OHSS in the agonist trigger group, although significantly lower than in the hCG trigger group, was surprisingly high (5%). It is likely that the exceptionally high rate of OHSS among IVF patients triggered with a GnRH agonist trigger in the study by Elgindy et al. (16) stems from the addition of hCG on the day of OPU as well as from the fact that all these patients underwent embryo transfer and therefore fetal hCG may have contributed to the higher rates of late OHSS. In contrast, a small retrospective study by Safrai et al. (17) reported similar OPR and LBR and no OHSS following MVP or oral dydrogesterone LPS in GnRH triggered cycles. A recent meta-analysis concluded that only when GnRH agonist trigger is given solely, with neither a concomitant low dose hCG nor with post agonist trigger hCG luteal support, is the risk for OHSS totally eliminated (18).

### LPS in frozen embryo transfers

**Figure 2:**
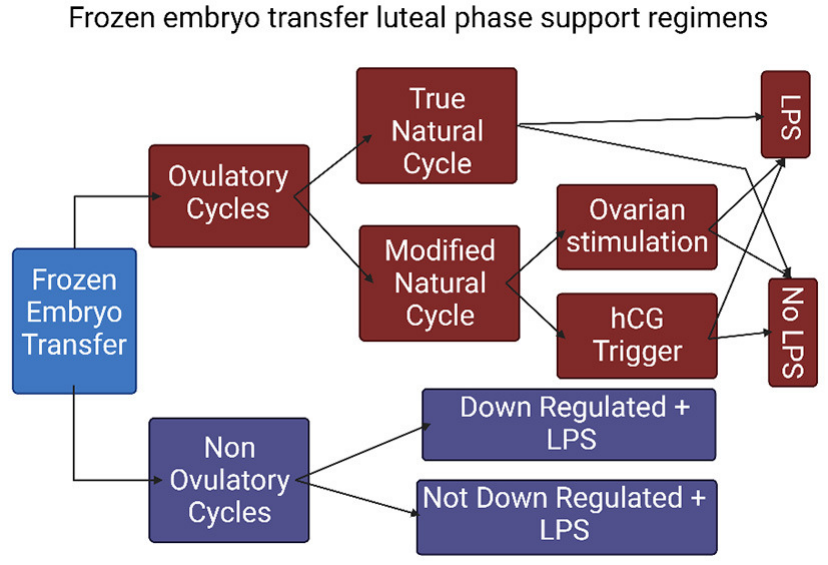
The types of frozen embryo transfer protocols. (FET, frozen embryo transfer; NC, natural cycle; COH, controlled ovarian hyperstimulation; tNC, true natural cycle; mNC, modified natural cycle; LPS, luteal phase support).

In frozen thawed embryo transfer (FET), unlike fresh ET, endometrial preparation is not secondary to ovarian stimulation and often occurs in the absence of a functional corpus luteum. In evaluating the contribution of LPS in FET one must take into consideration the type of endometrial preparation and the agents and routes of administration, as well as the use of an ovulation trigger. There are several types of endometrial preparation protocols, mainly: (1) true natural cycle (tNC) in which a thawed embryo is transferred following spontaneous ovulation; (2) modified natural cycle (mNC) in which ET occurs following hCG-triggered ovulation; and (3) artificial cycle (AC) also known as hormone replacement therapy (HRT) as well as programed FET protocol, in which the source of estrogen and progesterone in the absence of a CL is exclusively exogenous (Figure 2). Therefore, HRT protocols have two main objectives. The first is to induce adequate endometrial proliferation reaching satisfactory endometrial thickness. The second is to prevent follicle development and spontaneous premature ovulation that may skew the length of progesterone exposure and shift the WOI. To address these, HRT protocols are often based on early follicular administration of a high dose of estradiol that concomitantly induces endometrial proliferation as well as suppression of pituitary FSH secretion and the resulting follicular recruitment and development. Alternatively, HRT protocols may include a GnRH agonist to suppress follicle development. These protocols are often referred to as HRT with suppression. The contribution of LPS to treatment outcome in different FET preparation regimens was reviewed in a meta-analysis by Yarali et al. (19) and will be detailed in the following sections.

### LPS in true natural cycles

The need for LPS in a true natural cycle in which ET is synchronized with an ovulatory cycle is not obvious. A recently published meta-analysis focused on the effect of LPS in tNC (20). The analysis included eight studies, two of which used hCG for luteal support, and the remaining six studies used progesterone LPS. While hCG luteal support appeared to be non-beneficial, progesterone LPS showed a significant benefit both in clinical pregnancy rate (OR 1.48, 95 CI 1.14–1.94) and LBR (OR 1.67, 95% CI 1.19–2.36). These effects remained significant even when restricting the analysis to include only randomized trials. A possible explanation is that since in natural cycles, invariably, there is a single corpus lutem. The potential for increasing serum progesterone by augmenting its stimulation with hCG is limited by its capacity. On the other hand, the administration of external progesterone in natural cycle can result in higher serum progesterone reflecting the dose and route of administration.

### LPS in modified natural cycles

A mNC protocol includes an hCG trigger to induce ovulation that may be preceded by administration of aromatase inhibitors, clomiphene citrate, or gonadotropins to promote follicle development. Occasionally this results in the formation of more than one CL, emphasizing the need for LPS. A recently published small RCT comparing mNC with vs. without LPS showed similar treatment outcomes (21). In contrast, a retrospective study similarly comparing mNC with vs. without LPS found a significantly lower miscarriage rate and a higher LBR in the group receiving LPS (22).

Despite the logical distinction regarding the need for LPS between tNC an mNC protocols, a RCT by Mackens et al. (23) failed to show any difference in outcome between tNC and mNC in patients not prescribed with LPS. In a meta-analysis, pooled estimates for clinical pregnancy and LBR between tNC and mNC were not statistically significant whether LPS was used or not (19). The discrepancy between these findings may stem from the inclusion of ovarian stimulation in addition to hCG trigger in part of the mNC cycles.

### LPS in artificial cycles

Since ACs are inherently non-ovulatory and therefore lack a functioning CL, there is an essential need for LPS to synchronize the endometrium and set the WOI. Although, understandably, no studies compared AC FETs with vs. without LPS, multiple studies looked at various aspects of LPS in ACs. Progesterone vehicle and composition, routes of administration, dosage, serum concentrations, timing of progesterone treatment, and the inclusion of adjuvant agents have been the focus of multiple studies.

Yarali et al. (19) analyzed studies that compared both tNCs and mNCs with ACs that either included GnRH suppression or not. Pooled estimates for clinical pregnancy and LBR between tNC and AC without suppression demonstrated a statistically significant difference in favor of tNC (OR 1.46, 95 % CI 1.07–1.99) for clinical pregnancy rate but only a non-significant trend in LBR. However, in a sub-analysis including only tNC with LPS, statistical significance was not achieved while the trend remained positive.

Pooled estimates for clinical pregnancy and LBR between tNC and AC with suppression demonstrated a statistically significant difference in favor of AC with suppression (OR 0.73, 95% CI 0.56–0.95) for LBR but only a non-significant trend for clinical pregnancy. However, in a sub-analysis that included only tNC patients with LPS, statistical significance was achieved for both clinical pregnancy and LBR (OR 0.65; 95% CI 0.48–0.87 and 0.62; 95% CI 0.44–0.87; respectively). Of note, these results were derived from a single retrospective study (24).

Pooled estimates for clinical pregnancy and LBR between mNC with LPS vs. AC without suppression demonstrated no statistically significant difference (19).

#### Progesterone routes of administration in frozen embryo transfers

The route of progesterone administration in ART treatments is a topic of much debate. Although daily IM administration of natural progesterone dissolved in oil is considered the gold standard, it is associated with severe discomfort, and rarely, with sterile abscesses and risk of secondary infection (25). Recently, administering progesterone IM once every 3 days in combination with vaginal progesterone was shown to be non-inferior to the daily administration regimen, thereby reducing the level of discomfort (26). However, the vaginal route of administration may be associated with local irritation and lower and inconsistent serum concentration of progesterone (27).

A new preparation of aqueous progesterone that can be administered subcutaneously recently became available (28–30). Using this SC preparation, two non-inferiority RCTs compared 25 mg SC progesterone to micronized vaginal progesterone (MVP) in fresh ET and showed similar efficacy (28, 31). In addition, a retrospective study comparing SC to IM progesterone in AC FETs demonstrated similar outcomes (30). Several retrospective studies compared IM vs. vaginal administration. However, results were mixed, either supporting the use of IM progesterone over MVP (32, 33) or showing no significant differences (34).

Two recently published RCTs compared pregnancy rates in FETs protocols with vaginal progesterone administration for luteal support vs. IM injections. Both studies demonstrated that IM administration was associated with a significantly higher serum progesterone concentration (26, 35). The study by Davine et al. included a larger sample of 1125 FET cycles and therefore was powered to demonstrate not only increased progesterone levels but also higher LBR in the daily IM arm. The study design also included a combined MVP and IM arm (daily MVP and IM injection every 3 days) which was associated with similar LBR (26). In fact, an interim analysis resulted in discontinuation of the MVP arm due to the poorer outcome. In this study, the clinical pregnancy rate, total pregnancy loss, and LBR were 57, 56, and 39%, and 33, 26, and 52%, and 46, 48, and 29%, for the IM progesterone, combined IM and vaginal progesterone, and MVP only groups, respectively (26). It therefore appears that despite the discomfort associated with IM progesterone, in the context of FET it provides a superior outcome compared to vaginal progesterone only.

#### Timing of progesterone administration in frozen embryo transfers

As FETs include several types of protocols that differ in the way progesterone is introduced, the use of a theoretical OPU (tOPU) has been suggested as the point of reference to define the optimal time of LPS initiation (36) (Figure 1). Since FET cycles do not include an OPU, a theoretical OPU is defined as the age of the embryo in days at the time of vitrification minus one day. Moreover, as the exact timing of exposure to an effective dose of progesterone is critical in setting in motion the chain of events that lead to a synchronized WOI, it was essential to analyze trends in serum progesterone and decidual histology, as well as rates of implantation in ovulatory and non-ovulatory FETs, to determine the sequence of these events. Mackens et al. (36) summarized several comparative studies that examined alternative onsets of LPS in AC, tNC, and mNC frozen embryo transfer protocols. The authors found that in non-ovulatory HRT cycles, similarly to fresh ET, in order to achieve optimal rates of implantation and lowest risks of early pregnancy loss, LPS should be initiated on the day of tOPU. Hence, cleavage stage embryos should be transferred on the fourth day of LPS and blastocysts on the sixth day. The authors also addressed the timing of ET following an LH surge in tNCs vs. the timing post hCG trigger in mNCs. In tNCs ovulation occurs spontaneously 24 to 56 h post LH surge and is also preceded by a preovulatory rise in serum progesterone (37). In contrast, in mNCs the preovulatory rise in serum progesterone may not occur and ovulation is artificially triggered with hCG. Together with data on optimal pregnancy rates following intrauterine insemination post LH surge vs. HCG trigger (38), it was deduced the time interval between the LH surge and the tOPU would be 1 day shorter than the interval post hCG trigger and tOPU in mNCs (36). A retrospective study by Noble et al. (39) presented at the ESHRE conference in 2020, showed data on pregnancy rate in tNC before and after a change in ET policy. The change in policy led to a shortening of the interval from LH surge to ET from 7 to 6 days in tNC. This change from ET on LH+7 to LH+6 was associated with higher adjusted ongoing pregnancy rate (OPR) beyond 24 weeks (45 vs. 29%, aOR 2.13 95%CI 1.44–3.14, *p* < 0.0001). Meanwhile, the AC FET OPR at the corresponding time periods was similar (38.9 vs. 43%, aOR 0.90, 95%CI 0.70–1.16, *p* = 0.41), implying that the change in OPR in the tNC was due to the adjustment of ET to LH+6.

#### Dosing of progesterone support in frozen embryo transfers and LPS personalization

Intramuscular injections of progesterone have been traditionally associated with higher serum progesterone compared to MVP (26). As several recent publications suggested that IM progesterone results in a higher serum progesterone and OPR in FETs (26, 32, 33) the question still remains as to the optimal range of serum progesterone for achieving a pregnancy in FETs (10). A large retrospective study by Yovich et al. (40) correlated serum progesterone on the day of FET with CPR and LBR. The results support an optimal mid luteal progesterone range (70–99 nmol/l), whereas levels below and above this range were associated with significant decrease in CPR. The effect of serum progesterone concentration was independent of embryo grading, body mass index or the woman's age, either at vitrification or at FET. Gao et al. (41), examined whether administration of 40 mg IM progesterone to women with low serum progesterone (<10 ng/ml) measured on the day of FET may alter the expected suboptimal outcome. This large sample retrospective study showed that despite progesterone augmentation, the low serum progesterone group still had a significantly lower CPR (aRR 0.81 95%CI 0.68–0.96) and LBR (aRR 0.84 95%CI 0.70–1.0). The partial response to correction of low serum progesterone on the day of the transfer could represent an abnormal secretory transition and synchronization due to the low progesterone prior to the correction. Labarta et al. reported on a retrospective study in which patients diagnosed with low serum progesterone (<9.2 ng/ml) while on the standard LPS (400 mg of micronized vaginal progesterone twice daily) on the day of a frozen thawed blastocyst ET where either given an additional SC progesterone 25 mg, or not. The authors reported on a higher LBR in the group of patients supplemented with SC progesterone (44.9% vs. 37.3, OR 1.37; 95% CI 1.06–1.78) (9). However, since the definition of low serum progesterone ranges between 9.2 and 22 ng/ml (29.2 to 70 nmol/L), and the nature of the intervention used (IM progesterone vs. SC, 25–40 mg) in these studies differs, a well-designed large RCT is needed to assess the value of personalization.

## Conclusions

**Figure 3:**
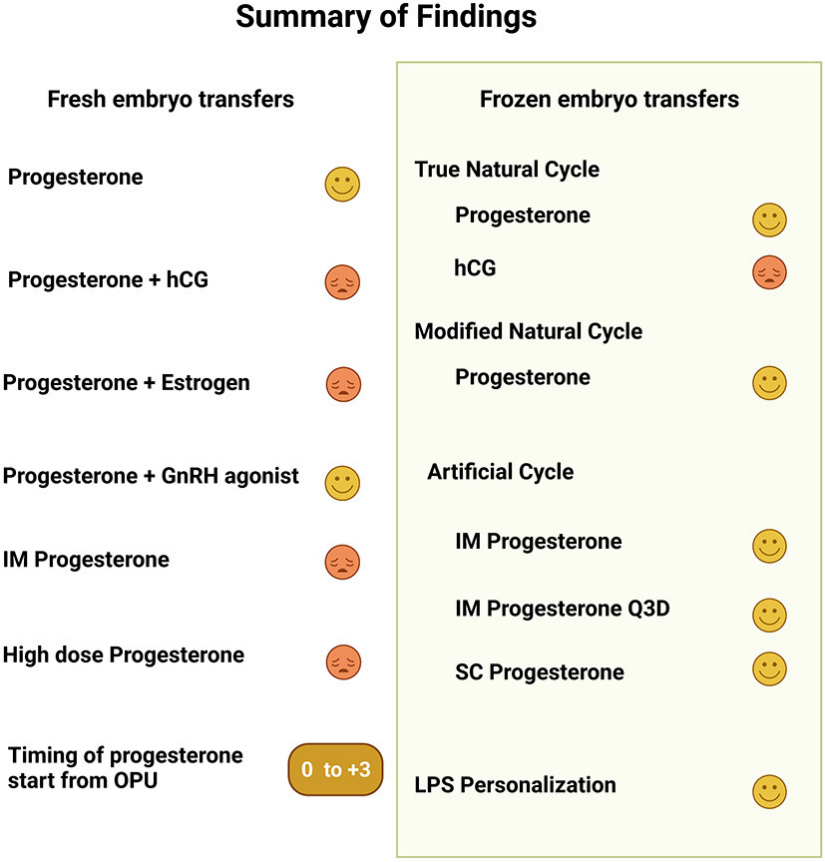
This a graphic summary of the findings presented in the article regarding luteal phase support in fresh and frozen embryo transfers. For each topic the symbol 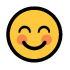 represents positive change in outcome while 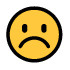 represents either no change or a poorer outcome.

Figure 3 provides a graphic summary of the main findings. The currently available data support the use of progesterone for LPS over hCG. In fresh ETs the addition of hCG or estrogen to progesterone LPS was non beneficial, yet a single or repeated doses of GnRH agonist were advantageous. In fresh ETs no route of progesterone administration provided a superior outcome. In frozen embryo transfers the inclusion of LPS seems to result in improved outcomes, both in ovulatory and programed cycles. Recent studies provide compelling evidence to an advantage for the IM route of progesterone administration over the vaginal approach. However, in both fresh and frozen ETs, a higher serum progesterone does not guarantee an optimal outcome. Finally, in ovulatory FET cycles the interval to embryo transfer should be a day shorter following LH surge compared to hCG trigger.

## Author contributions

All authors listed have made a substantial, direct, and intellectual contribution to the work and approved it for publication.

## Conflict of interest

The authors declare that the research was conducted in the absence of any commercial or financial relationships that could be construed as a potential conflict of interest.

## Publisher's note

All claims expressed in this article are solely those of the authors and do not necessarily represent those of their affiliated organizations, or those of the publisher, the editors and the reviewers. Any product that may be evaluated in this article, or claim that may be made by its manufacturer, is not guaranteed or endorsed by the publisher.
