# Supplementary material for: Luteal phase support in fresh and frozen embryo transfers
Source: Front Reprod Health. 2022 Jul 19;4:919948. doi: 10.3389/frph.2022.919948 (PMC9580718; doi:10.3389/frph.2022.919948)
Supplement: Supplementary file 1 [file Table_1.DOCX]

**List of RCTs & Meta-analyses included in the manuscript:**

**Fresh Transfers**

**Need for Luteal support**

Beckers NG, et al. Nonsupplemented luteal phase characteristics after the administration of recombinant human chorionic gonadotropin, recombinant luteinizing hormone, or gonadotropin-releasing hormone (GnRH) agonist to induce final oocyte maturation in in vitro fertilization patients after ovarian stimulation with recombinant follicle-stimulating hormone and GnRH antagonist cotreatment. J Clin Endocrinol Metab. 2003;88(9):4186-92. doi:10.1210/jc.2002-021953.

Hill MJ et al. Progesterone luteal support after ovulation induction and intrauterine insemination: a systematic review and meta-analysis. Fertil Steril. 2013;100(5):1373-80. doi:10.1016/j.fertnstert.2013.06.034.

**Progesterone Luteal support**

**Progesterone Vs Placebo**

Van der Linden M et al. Luteal phase support for assisted reproduction cycles. Cochrane Database Syst Rev. 2015(7):CD009154. doi:10.1002/14651858.CD009154.pub3.

**Progesterone Vs Progesterone & hCG**

Van der Linden M et al. Luteal phase support for assisted reproduction cycles. Cochrane Database Syst Rev. 2015(7):CD009154. doi:10.1002/14651858.CD009154.pub3.

**Progesterone Vs Progesterone & Estrogen**

Van der Linden M et al. Luteal phase support for assisted reproduction cycles. Cochrane Database Syst Rev. 2015(7):CD009154. doi:10.1002/14651858.CD009154.pub3.

**Progesterone Vs Progesterone & GnRH agonist**

Van der Linden M et al. Luteal phase support for assisted reproduction cycles. Cochrane Database Syst Rev. 2015(7):CD009154. doi:10.1002/14651858.CD009154.pub3.

**Progesterone routs of administration & dose**

Van der Linden M et al. Luteal phase support for assisted reproduction cycles. Cochrane Database Syst Rev. 2015(7):CD009154. doi:10.1002/14651858.CD009154.pub3.

Gari S, Al-Jaroudi D. Adding Weekly Intramuscular Progesterone to a Twice Daily Vaginal Progesterone Capsule for Luteal Phase Support in IVF/ICSI Cycles Results in Similar Live Birth Rates. JBRA Assist Reprod. 2021. doi:10.5935/1518-0557.20210040.

Nho EJ et al. Efficacy of dual progesterone administration (intramuscular and vaginal) for luteal support in fresh day 3 or day 4 embryo transfer cycles. Clin Exp Reprod Med. 2020;47(3):227-32. doi:10.5653/cerm.2020.03489.

Labarta E et al. Individualized luteal phase support normalizes live birth rate in women with low progesterone levels on the day of embryo transfer in artificial endometrial preparation cycles. Fertil Steril. 2022;117(1):96-103. doi:10.1016/j.fertnstert.2021.08.040.

Thomsen LH et al. The impact of luteal serum progesterone levels on live birth rates-a prospective study of 602 IVF/ICSI cycles. Hum Reprod. 2018;33(8):1506-16. doi:10.1093/humrep/dey226.

**Timing of Progesterone administration**

Connell MT et al. Timing luteal support in assisted reproductive technology: a systematic review. Fertil Steril. 2015;103(4):939-46 e3. doi:10.1016/j.fertnstert.2014.12.125.

Sohn SH et al. Administration of progesterone before oocyte retrieval negatively affects the implantation rate. Fertil Steril. 1999;71(1):11-4. doi:10.1016/s0015-0282(98)00404-x.

Williams SC et al. Delaying the initiation of progesterone supplementation results in decreased pregnancy rates after in vitro fertilization: a randomized, prospective study. Fertil Steril. 2001;76(6):1140-3. doi:10.1016/s0015-0282(01)02914-4.

**Progesterone personalization**

Thomsen LH et al. The impact of luteal serum progesterone levels on live birth rates-a prospective study of 602 IVF/ICSI cycles. Hum Reprod. 2018;33(8):1506-16. doi:10.1093/humrep/dey226.

**LPS post GnRH agonist trigger**

Elgindy EA et al. Towards an optimal luteal support modality in agonist triggered cycles: a randomized clinical trial. Hum Reprod. 2018;33(6):1079-86. doi:10.1093/humrep/dey054

Ioannidou PG et al. How frequent is severe ovarian hyperstimulation syndrome after GnRH agonist triggering in high-risk women? A systematic review and meta-analysis. Reprod Biomed Online. 2021;42(3):635-50. doi:10.1016/j.rbmo.2020.11.008.

**Frozen Transfers**

**LPS in true natural cycles**

Mizrachi Y et al. Should women receive luteal support following natural cycle frozen embryo transfer? A systematic review and meta-analysis. Hum Reprod Update. 2021;27(4):643-50. doi:10.1093/humupd/dmab011.

**LPS in modified natural cycles**

Horowitz E et al. A randomized controlled trial of vaginal progesterone for luteal phase support in modified natural cycle - frozen embryo transfer. Gynecol Endocrinol. 2021;37(9):792-7. doi:10.1080/09513590.2020.1854717.

Mackens S et al. To trigger or not to trigger ovulation in a natural cycle for frozen embryo transfer: a randomized controlled trial. Hum Reprod. 2020;35(5):1073-81. doi:10.1093/humrep/deaa026.

Yarali H, Polat M, Mumusoglu S, Yarali I, Bozdag G. Preparation of endometrium for frozen embryo replacement cycles: a systematic review and meta-analysis. J Assist Reprod Genet. 2016;33(10):1287-304. doi:10.1007/s10815-016-0787-0.

**LPS in artificial cycles**

Yarali H, Polat M, Mumusoglu S, Yarali I, Bozdag G. Preparation of endometrium for frozen embryo replacement cycles: a systematic review and meta-analysis. J Assist Reprod Genet. 2016;33(10):1287-304. doi:10.1007/s10815-016-0787-0.

**Progesterone routs of administration & dose**

Devine K et al. Intramuscular progesterone optimizes live birth from programmed frozen embryo transfer: a randomized clinical trial. Fertil Steril. 2021;116(3):633-43. doi:10.1016/j.fertnstert.2021.04.013.

Baker VL et al. A randomized, controlled trial comparing the efficacy and safety of aqueous subcutaneous progesterone with vaginal progesterone for luteal phase support of in vitro fertilization. Hum Reprod. 2014;29(10):2212-20. doi:10.1093/humrep/deu194.

Lockwood G et al. Subcutaneous progesterone versus vaginal progesterone gel for luteal phase support in in vitro fertilization: a noninferiority randomized controlled study. Fertil Steril. 2014;101(1):112-9 e3. doi:10.1016/j.fertnstert.2013.09.010.

Hershko Klement A et al. Intramuscular versus Vaginal Progesterone Administration in Medicated Frozen Embryo Transfer Cycles: A Randomized Clinical Trial Assessing Sub-Endometrial Contractions. Gynecol Obstet Invest. 2018;83(1):40-4. doi:10.1159/000475464.

**Timing of Progesterone administration**

Mackens S et al. Frozen embryo transfer: a review on the optimal endometrial preparation and timing. Hum Reprod. 2017;32(11):2234-42. doi:10.1093/humrep/dex285.
